# Crosstalk among proteome, acetylome and succinylome in colon cancer HCT116 cell treated with sodium dichloroacetate

**DOI:** 10.1038/srep37478

**Published:** 2016-11-22

**Authors:** Danxi Zhu, Lidan Hou, Bin Hu, Hang Zhao, Jie Sun, Jianhua Wang, Xiangjun Meng

**Affiliations:** 1 Department of Gastroenterology, Shanghai Ninth People’s Hospital, Shanghai Jiao Tong University School of Medicine, Shanghai, 200011, P.R. China; 2 Department of Gastroenterology, Shanghai General Hospital, Shanghai, 200060, P.R. China; 3 Department of Gastroenterology, Suzhou Science & Technology Town Hospital, Suzhou, 215000, P.R. China; 4 Fudan University Shanghai Cancer Center, Cancer institute, Shanghai, 200230, P.R. China

## Abstract

Protein lysine acetylation and succinylation play important regulatory roles in cells, both of which or each other has a close relationship. Dichloroacetate (DCA), a well-known pyruvate dehydrogenase kinase (PDK) inhibitor, has the potential to be used as anti-cancer drugs for several tumors including colorectal cancer. However, little is known about the potential mechanism of DCA-based cancer therapy by protein posttranslational modifications (PTM) including global proteome, acetylome and succinylome. Here the combinations with stable isotope labeling (SILAC), antibody affinity enrichment and high resolution LC-MS/MS analysis were performed in human colon cancer HCT116 cells. The quantifiable proteome was annotated using bioinformatics. In total, 4,518 proteins, 1,436 acetylation sites, and 671 succinylation sites were quantified, respectively to DCA treatment. Among the quantified acetylated sites, 158 were with increased level (quantification ratio >1.5) and 145 with decreased level (quantification ratio <0.67). Meanwhile, 179 up-regulated and 114 down-regulated succinylated sites were identified. The bioinformatics analyses initially showed acetylation and succinylation were involved in a wide range of cellular functions upon DCA-based anti-cancer effects. Notably, protein-protein interaction network analyses demonstrated widespread interactions modulated by protein acetylation and succinylation. Taken together, this study may shed a light on understanding the mechanism of DCA-based cancer treatment.

Protein posttranslational modifications (PTMs), which can alter structural, conformational and physicochemical properties of proteins, are key cellular events involved in many biological processes^1^,^2^. Among all the amino acids, lysine is a frequent target to be modified, which can be subjected to a variety of PTMs^3^. With the advances in high-resolution mass spectrometry (MS) and antibody-based affinity enrichment of lysine (Lys) residues, a lot of novel PTMs have been identified, such as ubiquitylation, butyrylation, succinylation, and glutarylation.

The acetylation of Lys residues in proteins has a role in transferring acetyl moiety from acetyl-CoA to its amino groups. Early studies of Lys acetylation mainly focused on histones and other transcription factors in the nucleus^4^,^5^,^6^. However, they have been found recently to occur in almost every compartment of a cell, such as the cytoplasm and mitochondria, and play a major role in metabolic regulation, including glycolysis, tricarboxylic acid (TCA) cycle, fatty acid metabolism and so on^7^,^8^. Succinylation refers to the transfer of succinyl group from the succinyl donor succinyl-CoA to the ε-amino group of specific lysine residue of the target protein, which is also considered to occur frequently like acetylation in several cellular events^9^. Most of the identified succinylated proteins are enzymes involved in kinds of metabolic pathways, which are important for the regulation of central metabolism such as fatty acid metabolism, amino acid degradation. It was reported that acetylation and succinylation were closely linked to central carbon metabolism^10^. Brian *et al*.^11^ revealed that majority of succinylation sites in bacteria, yeast, and mouse liver were also acetylated at the same position^11^. Yuta Mizuno *et al*.^12^ found that lysine acetylation and succinylation target most enzymes in central carbon metabolic pathways that are directly linked to glutamate production, and the extent of modification changed in response to glutamate overproduction^12^.

Dichloroacetate (DCA), an inhibitor of pyruvate dehydrogenase kinase (PDK), can reverse the Warburg effect and induce apoptosis in tumor cell by increasing the flux of pyruvate into the mitochondria and promoting glucose oxidation^13^. The increasing evidence in preclinical *in vitro* and *in vivo* indicates that DCA may be a promising selective anti-cancer agent. However, the potential mechanism of DCA-based cancer treatment is still unclear.

Given the widespread regulatory role of PTMs and the close relationship between acetylation and succinylation, we are interested to explore the regulative mechanism of DCA treatment by PTM. Here, an integrated system of SILAC labeling, HPLC fractionation and affinity enrichment followed by high resolution LC-MS/MS analysis were performed for the quantitative comparison of the global proteome, acetylome and succinylome in HCT116 cells with or without DCA treatment. Intensive bioinformatics analyses were then used to annotate those quantifiable modified targets in response to DCA treatment. Our studies indicate that the approach described above is powerful for identification of modified proteins and peptides. Therefore, the results provided a novel insight into DCA treatment on colon cancer cells.

## Results

### Analysis of lysine acetylation, and succinylation sites in HCT116 cells stimulated by DCA

**Figure 1:**
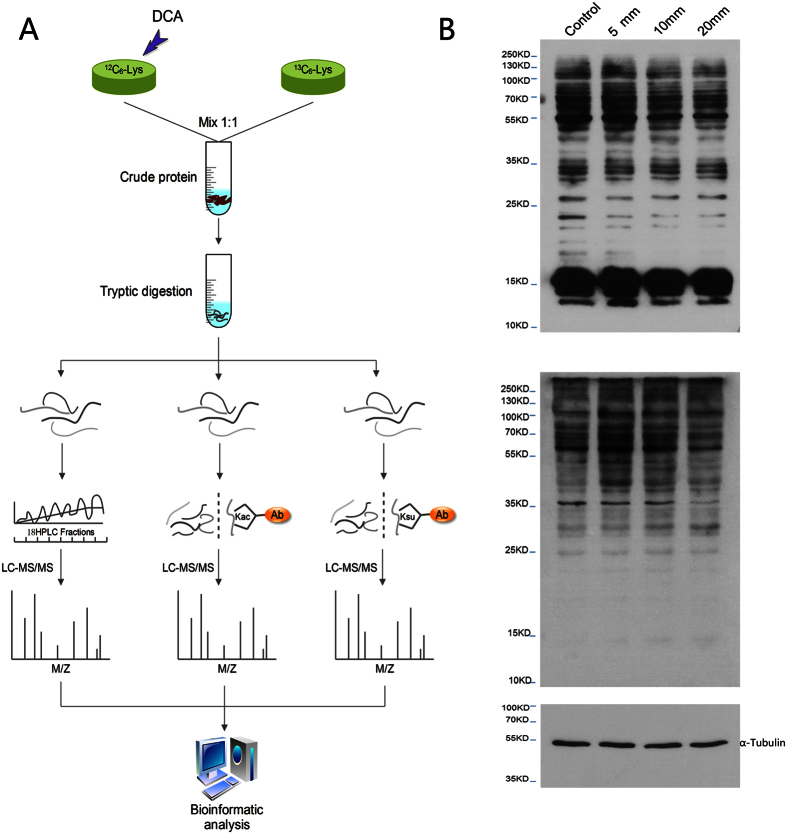
Assaying DCA-based cancer treatment by PTM. (**A**) The analytical strategy and method for DCA-responsive quantitative profiling of global proteome, acetylome and succinylome in HCT116 cell line^33^,^34^ (The Edraw Max V7.3 software is used to create the map in (**A**), https://www.edrawsoft.com/term-conditions.php). (**B**) Western blotting with anti-acetyl lysine antibody (up) and anti-succinyl lysine antibody (down).

To identify the profiles of the global proteome and lysine acetylome, succinylome, we carried out a 6-step workflow: (1) stable isotope labeling of HCT116 cells with or without DCA stimulation by SILAC; (2) protein extraction and trypsin digestion to yield peptides; (3) HPLC fractionation; (4) affinity enrichment of lysine acetylated and succinylated peptides; (5) LC-MS/MS analysis was used to identify the enriched peptides; (6) database search and bioinformatic analysis (Fig. 1).

**Figure 2:**
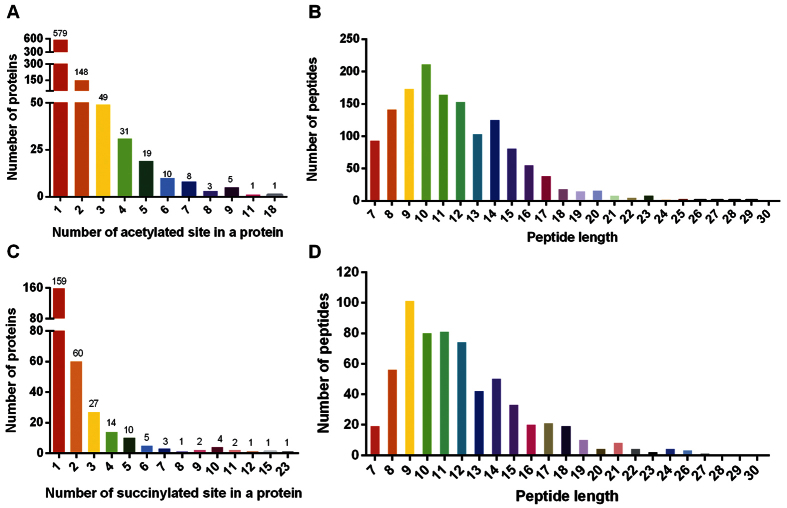
The distribution of acetylated proteins and succinylated proteins. (**A**) Distribution of acetylated proteins based on their number of modified sites. (**B**) Distribution of acetylated peptides based on their length. (**C**) Distribution of succinylated proteins based on their number of modified sites. (**D**) Distribution of succinylated peptides based on their length.

In this work, comparing cells with or without DCA treatment, 5,448 proteins identified and 4,518 proteins were quantified in aspects of global proteome. Among these, 244 proteins were increased and 269 proteins were decreased. In addition, we identified 1,484 lysine acetylation (Kac) sites in 860 proteins, among of which 1,436 Kac sites in 841 proteins were quantified. We also identified 680 lysine succinylation (Ksu) sites in 295 proteins, among of which 671 Ksu sites in 291 proteins were quantified. The identified acetylated and succinylated peptides showed different abundance depending on their lengths (Fig. 2B,D), match 860 acetylated and 295 succinylated proteins, respectively (Fig. 2A,C). Apparently, most of them were modified at sole site, consistent with the previous findings about post-translational modification^14^,^15^,^16^.

**Figure 3:**
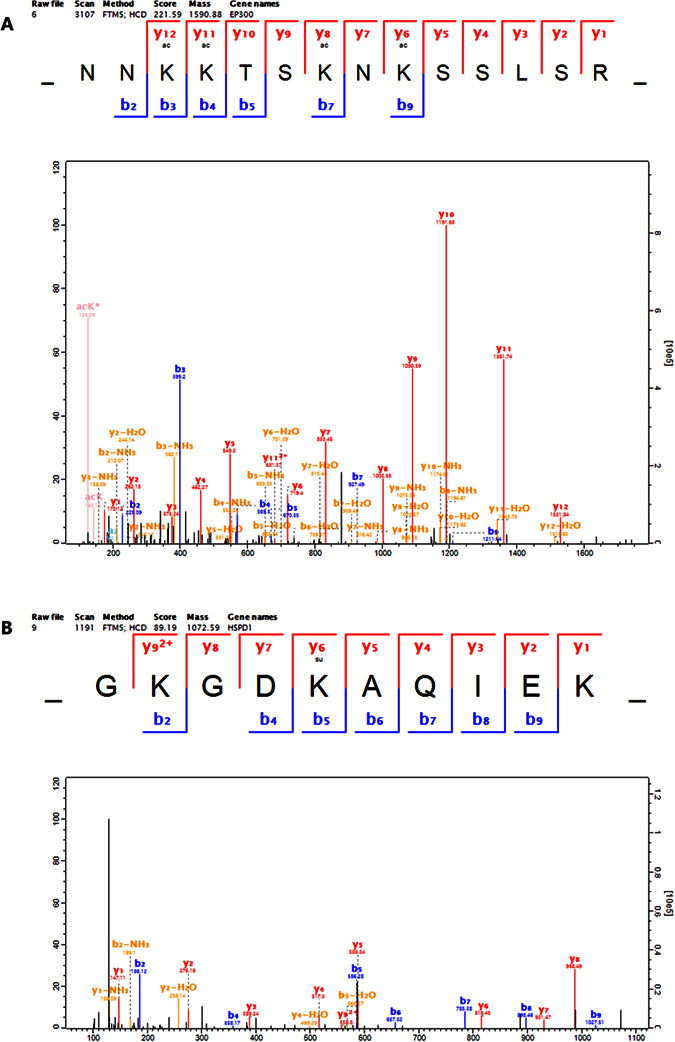
Representative MS/MS spectra of Histone acetyltransferase p300 acetylation (**A**) and 60 kDa heat shock protein succinylation (**B**).

Figure 3 shows the MS/MS spectra of two representative acetylated and succinylated proteins, histone acetyltransferase p300 and 60 kDa heat shock protein, respectively. Histone acetyltransferase p300 is encoded by gene *EP300*, and functions as histone acetyltransferase, which can regulate transcription via chromatin remodeling by acetylating four core histones. 60 kDa heat shock protein encoded by gene *HSPD1* is involved in mitochondrial protein import and macromolecular assembly. Meanwhile, it plays an important role in protein folding, refolding, and proper assembly of unfolded polypeptides generated under the stress conditions. The two proteins were obviously modified, implying their crucial function in response to DCA treatment.

### DCA-responsive global proteome

DCA has been reported as anti-cancer drug. It is therefore intrigue to assay the DCA-responsive proteome in HCT116 cells. Here, 5,448 proteins were identified and 4,518 proteins were quantified in the cells. Among them, 244 proteins were DCA-increased and 269 proteins were DCA-decreased (Supplementary Information Table S1) when we set quantification ratio of >1.5 as up-regulated threshold and <0.67 as down-regulated threshold. To elucidate the possible roles of these proteins, we performed four types of enrichment-based clustering analyses: Gene Ontology (GO) functional classification, KEGG pathway analysis, protein domain enrichment-based analysis, and protein complex analysis. The quantified proteomic dataset were divided into four quantiles according to L/H ratio to generate four quantiles: Q1 (0~25%), Q2 (25~50%), Q3 (50~75%), and Q4 (75~100%). Enrichment analyses were performed separately based on the quantiles.

We analyzed the quantifiable proteome dataset for three enrichment GO categories: biological process, molecular function, and cellular compartment (Figure S1A~C). For the molecular function analysis (Figure S1A), we found that proteins with increased L/H ratios were enriched in carboxylic ester hydrolase activity, and structural constituent of cytoskeleton. Proteins with helicase activity, pyrophosphatase activity, and hydrolase activity were enriched in Q1 and Q2. The cellular compartment analysis was presented in Figure S1B, indicating that many proteins located in keratin filament, extracellular space, and cytosol were enriched in quantiles Q3 and Q4 with high L/H ratios, which may suggest that might indicate DCA treatment have obvious influence on cytoarchitecture located in these places. The decreased proteins were mainly focused on chromosome and spindle, condensed chromosome, suggesting DCA may participate in cell cycle and mitosis.

In the biological process category (Figure S1C), proteins in Q3 and Q4 quantiles were enriched in epidermis development, cell differentiation, and other developmental processes. In contrast, proteins in Q1 and Q2 quantiles were enriched in cell cycle process, cell division, mitosis and organelle fission. These findings were consistent with results of the cellular compartment analysis. Taken together, DCA-responsive protein modifications have impacts on various processes such as replication, transcription, cell proliferation and so on. Importantly, the influence of DCA on cell developmental process and differentiation suggest that the potential role of DCA may act as an anti-cancer agent.

The Kyoto Encyclopedia of Genes and Genomes (KEGG) pathway analysis of the quantified proteins was also performed (Figure S1D). DCA-decreased proteins were mainly focused on cell cycle, DNA replication, steroid biosynthesis, pyrimidine metabolism, and p53 signaling pathway. While DCA-increased proteins were mainly involved in galactose metabolism, amino sugar and nucleotide sugar metabolism, as well as glycerolipid metabolism. It has been proved that DCA is the inhibitor of pyruvate dehydrogenase kinase (PDK)^17^. Besides, DCA has the ability to activate AMPK signaling pathway, which has a close relationship with glycolysis or gluconeogenesis^18^. The findings indicate that DCA has potential regulatory roles of participating in other kinds of glycometabolism.

Finally, the protein domain analysis indicated that the protein function domains involved in MCM N-terminal, mini-chromosome maintenance, and DNA-dependent ATPase were enriched in Q1 and Q2 quantiles, while protein domains participated in EGF-like conserved site, MCM N-terminal domain were enriched in Q3 and Q4 quantiles (Figure S1E).

### DCA-responsive acetylome

Acetylation is a dynamic and reversible PTMs, which can transfer acetyl moiety from acetyl-CoA or acetyl phosphate to lysine residues at ε-amino groups in proteins^18^,^19^. Lysine acetylation was found in histones^20^, and in non-histones^21^, which has diverse functions like regulation of gene transcription, cell cycle, apoptosis, metabolic flux and so on^15^,^22^. It has been reported that DCA may have a relationship with acetylation^23^, however the direct evidence needs to be further investigated.

Here, the acetylation level of proteins in response to DCA stimulation was investigated by the combination of SILAC labeling, lysine acetylation antibody enrichment and LC-MS/MS analysis. Notably, 1,484 lysine acetylation sites in 860 protein groups were assayed, among which 1,436 sites in 841 proteins were quantified (Table S2). When setting quantification ratio of >1.5 as up-regulated threshold and <0.67 as down-regulated threshold, 158 lysine acetylation sites in 116 proteins were quantified as up-regulated targets and 145 lysine acetylation sites in 112 proteins were quantified as down-regulated targets. To our knowledge, this is the first profiling of lysine acetylation dataset in HCT116 cells under DCA treatment.

**Figure 4:**
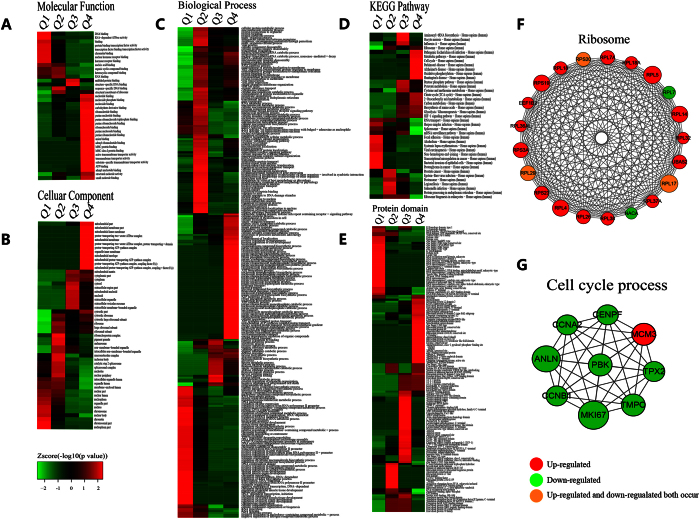
Clustering analysis of the quantified acetylome based on the functional enrichment. (**A**) Molecular function, (**B**) cellular compartment, (**C**) biological process, (**D**) KEGG pathway, and (**E**) protein domain. (**F**) Protein-protein interaction network of acetylated proteins clustered in ribosome. (**G**) Protein-protein interaction network of acetylated proteins clustered in cell cycle process.

The enrichment-based clusting analyses were then performed to compare the functions of corresponding DCA-responsive proteins. All these quantified acetylated proteins were divided into four quantiles (Q1~4) as described above, including Gene Ontology, KEGG pathway, protein domain and protein motif (Fig. 4A~E, Figure S2A,C).

In GO functional classification (Fig. 4A–C), the analysis of molecular function (Fig. 4A) showed that increased proteins involved in structural constituent of ribosome, structural molecule activity, ATPase activity were changed significantly, while the decreased proteins participated in nucleic acid binding, heterocyclic compound binding, and other bindings were severely affected. The results were consistent with the notion that DCA can lead to the inhibition of cell proliferation and cause cell death^24^. In cellular compartment (Fig. 4B), the increased proteins were mostly enriched in cytosolic ribosome, ribosome. In contrast, decreased proteins were mainly enriched in nucleolus, chromatin, and DNA bending complex. In the biological process category (Fig. 4C), nuclear-transcribed mRNA catabolic process, aromatic compound catabolic process, mRNA catabolic process were highly enriched, while the processes related to chromatin organization, regulation of skeletal muscle tissue development were significantly enriched. These results were consistent with the former analysis of molecular function, suggesting DCA may play a role in RNA and protein synthesis and therefore have influence on the growth of tumors.

To identify cellular pathways affected by DCA treatment, we then performed a pathway clustering analysis using KEGG (Fig. 4D). The results showed that ribosome, oxidative phosphorylation, carbon metabolism were the most prominent pathways enriched in quantiles with high L/H ratios, suggesting DCA functions as a regulatory factor of protein biosynthesis and energy metabolism. It is notable that these results were consistent with the notion DCA can alter the ‘Warburg Effect’ into normal oxidative phosphorylation and thus inhibit tumor growth. But in quantiles with low L/H ratios, protein expression in pathways of viral carcinogenesis, systemic lupus erythematosus, RNA transport were severely decreased in response to DCA stimulation.

As domain structure is one of the most important functional features of protein, we next analyzed the domain features of the proteins changed after DCA adding (Fig. 4E). We found that domains in proteins involved in Histone-fold, Histone core, Histone H2B were remarkably enriched upon DCA treatment in down-regulated quantiles, suggesting DCA have great influence on transcription. But Zinc finger, translation protein SH3-like domain, ribosomal protein L2 domain 2 were mainly enriched in up-regulated quantiles.

To determine if there were specific amino acids adjacent to acetylated lysines, we compared the sequences flanking acetylated sites by heat-map. A strong bias of amino acid sequence, namely, Tyrosine (Y), Phenylalanine (F), Histidine (H), Tryptophan (W), was found in our data. In addition, isoleucine (I) and arginine (R) were overrepresented in the two and four to five positions behind Kac sites (Figure S2A,C), suggesting aromatic groups were common to be modified by acetyltransferases. These results were consistent with former report using mycobacterium tuberculosis as test subjects^15^ and indicated lysine acetylating may be conserved and widespread.

Protein-protein interaction network of acetylome proteins was established by using Cytoscape software (Figure S4A and Fig. 4F~G). Two kinds of important pathways (ribosome and cell cycle process) were listed in Fig. 4F~G, indicating that proteins participated in cell cycle process have mostly been down-regulated except MCM3, which had been acetylated at multiple sites. The acetylation of this protein can inhibit the initiation of DNA replication and cell cycle progression. Meanwhile, MKI67, a nuclear protein that is associated with and may be necessary for cellular proliferation, was acetylated at multiple sites and their levels were down-regulated remarkably^25^. These findings can partly explain how DCA cause cell cycle arrest and inhibit cell proliferation.

### The correlation of DCA-responsive global proteome and acetylome

Upon the dataset of DCA-responsive proteome and acetylome, we performed the overlapping analysis between them. According to the quantitative results, 664 proteins were quantified both in proteome and acetylome, including a number of 1484 Kac sites. The quantitative ratios of proteome and acetylome were compared (Table S4) and the scatter diagram was shown in Figure S3A. The pearson’s correlation coefficient and the Spearman’s rank correlation coefficient were 0.235 and 0.236, respectively. The results demonstrate the global proteome and acetylome have little correlation with each other.

### DCA-responsive succinylome

**Figure 5:**
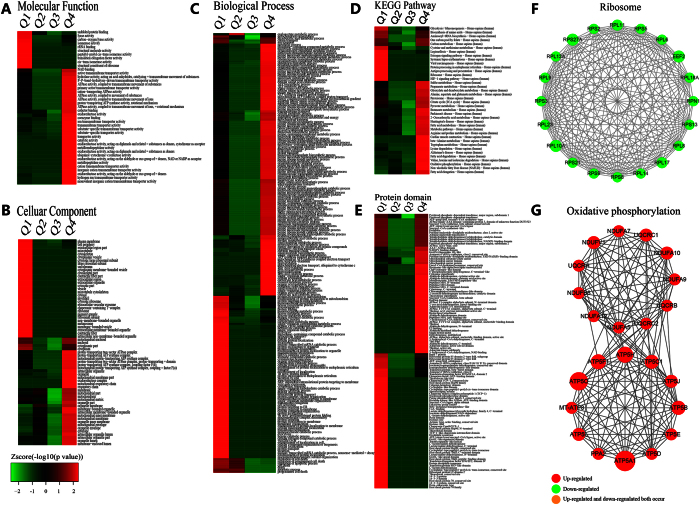
Clustering analysis of the quantified succinylome based on the functional enrichment. (**A**) Molecular function, (**B**) cellular compartment, (**C**) biological process, (**D**) KEGG pathway, and (**E**) protein domain. (**F**) Protein-protein interaction network of acetylated proteins clustered in ribosome. (**G**) Protein-protein interaction network of acetylated proteins clustered in oxidative phosphorylation.

The succinylome in HCT116 cells was identified by combining the SILAC, immuneaffinity enrichment by a high-specificity antibody (PTM Biolabs), and high-resolution mass spectrometry. We identified 680 lysine succinylation sites in 295 protein groups, among which 671 sites in 291 proteins were quantified. Among them, 179 lysine succinylation sites in 108 proteins were increased and 114 lysine succinylation sites in 71 proteins were decreased (Table S3). Then all the quantified succinylated proteins were divided into four quantiles (Q1~4) according to L/H ratios as described above. The clustering analyses included GO analysis, KEGG pathway analysis, protein domain analysis and motif analysis (Fig. 5A~E and Figure S2B,D).

For the molecular function analysis (Fig. 5A), we first found that increased proteins were highly enriched in oxidoreductase activity, hydrogen ion transmembrane transporter activity, substrate-specific transmembrane transporter activity, but for decreased proteins, they were mainly enriched in unfolded protein binding, protein binding and calcium ion binding. It was reported that plasma membrane oxidoreductase activity had a relationship with mitochondrial function and oxidative stress^26^, and excessive reactive oxygen species (ROS) could ultimately lead to ROS-mediated genomic instability and cancer^27^ as well as induce apoptosis. We propose that the enhanced oxidoreductase activity induced by DCA may be associated with its anti-cancer effect.

In the cellular compartment category (Fig. 5B), proteins located in the mitochondrial parts such as matrix, membrane, inner membrane were highly enriched in Q3 and Q4 quantiles, indicating that DCA-induced succinylation may have important roles in mitochondrial. However, proteins in Q1 and Q2 quantiles were mainly focused on pigment granule, melanosome and cytosol. The biological process of succinylation was displayed in Fig. 5C, which indicates that the up-regulated proteins were mainly enriched in small molecule metabolic process, oxidation-reduction process, cellular respiration. The decreased proteins were mainly concentrated on protein folding, cellular localization, and cellular component organization.

The KEGG pathway analysis for the succinylate proteins showed a number of important pathways (Fig. 5D). For proteins in Q1 and Q2 quantiles, the ribosome, viral carcinogenesis, TCA cycle, and glycolysis/gluconeogenesis pathways were highly enriched. In contrast, pathways of oxidative phosphorylation, Huntington’s disease, Alzheimer’s disease, and metabolism were highly enriched in Q3 and Q4 quantiles. These results implied succinylation has a close relationship with diseases and some metabolic pathways like oxidative phosphorylation, indicating succinylation may play an important role in DCA’s effects of turning ‘Warburg effect’ into normal oxidative phosphorylation. In addition, the pathways were highly enriched in Q3 and Q4 quantiles of acetylated proteins, implying the close relationship between these two PTMs. This was associated with the previous report, suggesting that acetylation and succinylation are closely overlapped^16^.

Figure 5E shows that protein domains involved in Histone-fold, Histone core, and Histone H2B were highly enriched in proteins with decreased Ksu sites, while several function domains (i.e., Zinc finger, LIM-type, Translation protein SH3-like, and Ribosomal protein L2) were highly enriched in proteins with increased Ksu sites.

Protein-protein interaction network of succinylated proteins was established by using Cytoscape software, and the global network of Ksu proteins was displayed in Figure S4B. The network indicated that succinylated proteins actively participated in ribosome and oxidative phosphorylation (Fig. 5F~G). Almost all succinylated proteins involved in ribosome were decreased and proteins involved in oxidative phosphorylation were increased. These findings imply that DCA-responsive succinylation play a significant role in protein synthesis and cellular energy metabolism.

To test whether conserved lysine succinylation motifs exist among lysine- acetylated proteins, we carried out an analysis of global succinylated proteins (Figure S2B,D). Three preferred sequence patterns were found as V*Ksu, I*Ksu, and R******Ksu. To our knowledge, V*Ksu and I*Ksu have already been observed to be existed in human, rat and *E. coli*, but neither of them were reported to be among the most common ones in the previous reports^11^. In addition, EK and Ksu*****K, two of the most common sequence patterns, were overrepresented in this study, suggesting the preferred sites for succinylation have some similarities even in different species.

### The correlation of DCA-responsive global proteome and succinylome.

We next assayed the features of DCA-responsive global proteome and succinylome. Totally, 295 quantified proteins were found in both of them, including 636 Ksu sites. The quantitative ratios of proteome and succinylome under DCA treatment were compared (Table S5), and the scatter diagram was shown in Figure S3B. To be accurate, the pearson’s correlation coefficient and the Spearman’s rank correlation coefficient were 0.255 and 0.292, respectively. The results demonstrate there is litte correlation between the global proteome and succinylome.

### The correlation of DCA-responsive acetylome and succinylome.

**Figure 6:**
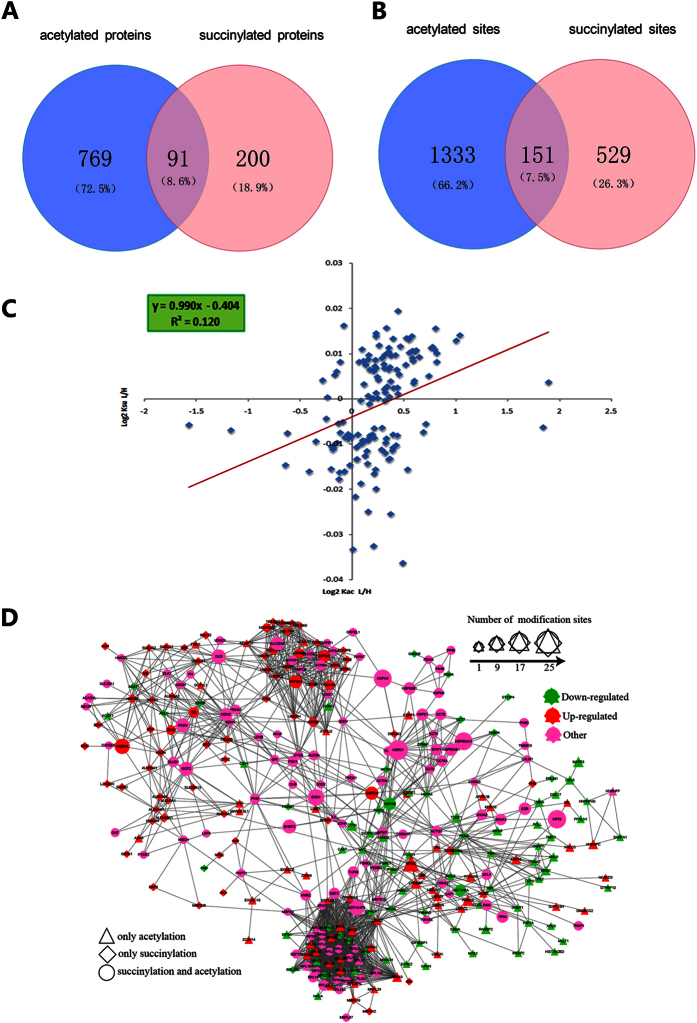
Correlation of global proteome, acetylome and succinylome. (**A**) Overlap between acetylated proteins and succinylated proteins. (**B**) Overlap between acetylated sites and succinylated sites. (**C**) Correlation of acetylome and succinylome. (**D**) Protein-protein interaction network between acetylated proteins and succinylated proteins.

It was reported that each PLM can crosstalk with at least one other PLM and the co-occurrences of different PLMs at the same site were abundant^28^. Here, the DCA-responsive acetylome and succinylome in HCT116 cells were assayed to investigate their relationships. 91 proteins were both acetylated and succinylated, but they were not modified at the same site exactly. Among them, there were 151 lysine sites were modified with both acetylation and succinylation (Fig. 6A,B). The correlation between the L/H ratios of acetylome and succinylome (Table S6), as well as the scatter diagram were shown in Fig. 6C. The Spearman’s correlation coefficient and Pearson’s was 0.347 and 0.478, respectively. This indicated that DCA-responsive patterns of acetylated and succinylated sites were positively related. Notably, some important proteins participated in glycolysis, the main pathway through which DCA regulates tumor cell metabolism, were acetylated and succinylated simultaneously, such as pyruvate kinase muscle (PKM), phosphoglycerate kinase 1 (PGK1), lactate dehydrogenase B (LDHB), and enolase 1 (ENO1). This implies that the two modifications are involved in DCA’s effect on HCT116 cells.

For assaying the relationship of acetylome and succinylome, the protein-protein interaction network was established. The global overview of network among Kac and Ksu proteins was performed by using Cytoscape (Fig. 6D). The complicated network demonstrates that the acetylated and succinylated proteins have a closed crosstalk, indicating that some important proteins underwent both acetylation and succinylation, such as dihydrolipoamide dehydrogenase (DLD), glyceraldehyde-3-phosphate dehydrogenase (GAPDH), and cytochrome C somatic (CYCS).

## Discussion

DCA has been reported to be a promising anti-cancer drug^29^, but its regulatory mechanisms in tumor cells are still unknown. In this study, a SILAC-based quantitative proteomic approach was used to investigate the anti-cancer effect of DCA on the proteome, acetylome and succinylome in HCT116 colon cancer cells.

The alternation of the whole proteome in HCT116 cells showed that proteins participated in cell cycle, cell division and proliferation apparently decreased, which linked gene expression levels with the DCA-induced anti-cancer effects. In addition, The KEGG pathway analysis of the quantified proteins showed that the increased proteins were highly enriched in galactose metabolism, amino sugar and nucleotide sugar metabolism, consistent with DCA’s role of regulating glucose metabolism.

The quantitative acetylome analysis revealed 1436 DCA-responsive Kac sites in HCT116 cells, which would be the most comprehensive Kac profiling in HCT116 cells. It indicated that the decreased proteins mainly located in nucleolus, chromatin, and DNA bending complex, participating in nucleic acid binding, which implys that DCA can inhibit DNA replication and regulate protein biosynthesis by lysine acetylation. In addition, we observed that some proteins encoded by important *myc* oncogene was significantly down-regulated.

In addition, among 680 lysine succinylation sites in 295 proteins, the DCA-increased proteins were mostly localized in mitochondrial and enriched in oxidoreductase activity, hydrogen ion transmembrane transporter activity, and substrate-specific transmembrane transporter activity. It has been reported that mitochondrial membrane potential and ROS production are dependent on the flux of electrons down the electron transport chain (ETC). Decreased entry of pyruvate would eventually result in decreased flux of electrons in the ETC and therefore reduced ROS production, contributing to the close of the redox-sensitive mitochondrial transition pore (MTP) and mitochondrial hyperpolarization which are considered to be closely related with tumor genesis^30^. These findings suggest that DCA may result in lysine succinylation and then increase the delivery of pyruvate into the mitochondria, which may help mitochondria-based glucose oxidation and mitochondrial depolarizing, thus returning the membrane potential towards the levels of normal cells, and exert its anti-cancer effect.

**Figure 7:**
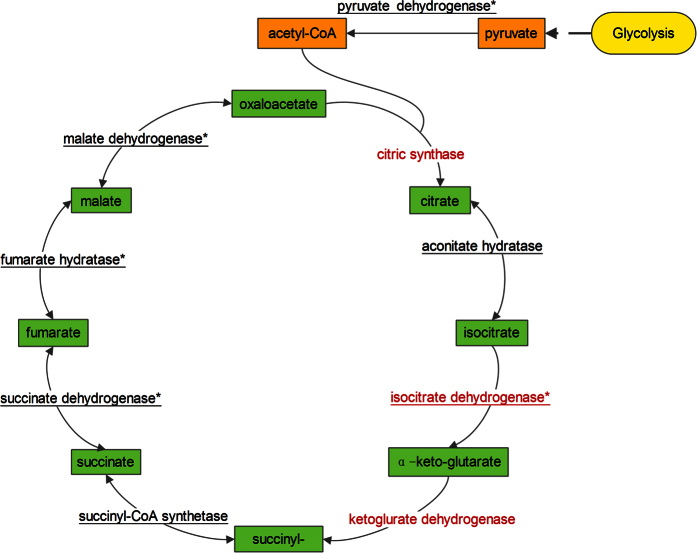
Acetylated and succinylated enzymes involved in TCA cycle. The enzymes identified as lysine-acetylated are marked with an asterisk. The enzymes identified as lysine-succinylated are underlined.

Considering the growing evidence suggests that lysine acetylation and succinylation may have a close relationship, we focused on several pathways undergoing the two modifications. Generally, the overlap between acetylome and succinylome were mainly related to oxidative phosphorylation, metabolic pathways, citrate cycle (TCA cycle), and carbon metabolism. Here, we discussed the TCA cycle in detail for its close relationship with DCA’s anti-cancer effect. It is well known that DCA can target PDK, which can connect glycolysis with TCA cycle by promoting catalyzing pyruvate to acetyl-CoA. We checked the modification of PDH (regulated by PDK) and eight enzymes participated in TCA cycle, including citric synthase, aconitate hydratase, isocitrate dehydrogenase, ketoglurate dehydrogenase, succinyl-CoA synthase, succinate dehydrogenase, fumarate hydrogenase, malate dehydrogenase in our dataset. Some of them were acetylated or succinylated (Fig. 7). However, PDK itself was not acetylated or succinylated in our study. Isocitrate dehydrogenase, which catalyzes the conversion of isocitrate to α-keto-glutarate, was involved both in acetylation and succinylation. There were a total of eight Ksu sites and 5 Kac sites on this enzyme. According to the previous report, mutagenesis of the succinylated lysine residues of this enzyme has a crucial role of the activity of the enzyme^31^. It is reasonable to suppose that the modifications described above will induce important changes of TCA cycle, especially when this step is irreversible and rate-limiting. But we didn’t find another two enzymes in citric synthase and ketoglurate dehydrogenase were acetylated or succinylated. In total, among the nine enzymes, seven were succinylated and five were acetylated in HCT116 cells after DCA treatment, respectively. These results suggest that the enzyme activity in metabolism has been positively regulated by acetylome and succinylome, thus mediating the drug effect.

Recent studies have reported that histone acetylation could induce the change of global proteome by epigenetics while non-histone acetylation of transcription factors could regulate proteome level through transcriptiome^32^,^33^. Our classification was not clear enough to show the direct relationship among global proteome, acetylome and succinylome. Obviously, to further confirm the view, more explicit classification and bioinformatic analysis should be done.

## Conclusions

Here we present a large-scale quantitative analysis of DCA-responsive global proteome, acetylome and succinylome in HCT116 cells by using high sensitivity mass spectrometry and bioinformatic analysis. In total, we identified DCA-responsive 860 Kac proteins and 295 Ksu proteins in cells. Our work provides a database that can be used to examine what effect DCA on cancer cells, drug resistance. Notably, the correlation of global proteome, acetylome and succinylome may expand our understanding of DCA’s anti-cancer effect. Although the underlying mechanism of acetylome and succinylome remains to be further explored, the current study shed a light on several biological processes like TCA cycle, glycolysis, and other functions.

## Methods and Materials

### HCT116 culture and SILAC labeling

Cells were grown to 80% confluence in high glucose Dulbecco’s modified Eagle’s medium (with glutamine and sodium pyruvate) (Pierce, MA, USA) containing 10% fetal bovine serum (Gibco, CA, USA) at 37 °C with 95% air and 5% CO_2_. Then cells were labeled with ‘light isotopic lysine’ (^12^C-Lysine) or ‘heavy isotopic lysine’ (^13^C-Lysine) using a SILAC Protein Quantitation Kit (Pierce, MA, USA) according to manufacturer’s instructions. After cells were expanded in SILAC media to our desired cell number (~5 × 10^8^), the ‘light’ labeled cells were then treated with 20 mM DCA (Sigma, MO, USA) and cultured for another 24 hours in SILAC media before harvesting.

### Protein extraction, Trypsin Digestion and HPLC fractionation

The harvested labeled cells were lysed with 2 × NETN buffer (200 mM NaCl, 100 mM Tris-Cl, 2 mM EDTA, 1.0% NP-40, pH 7.2) supplemented with 0.5% Triton X-100 on ice for 30 min. The unbroken cells or debris were removed after centrifuge at 20,000 g for 10 min at 4 °C. After concentration measurement, same amounts of proteins labeled with ‘heavy’ or ‘light’ were mixed and the crude proteins were precipitated. After washing twice with ice-cold acetone, the air-dried precipitate was dissolved in 100 mM NH_4_HCO_3_ (pH 8.0) and then digested with trypsin (Promega, WI, USA) at an enzyme-to substrate ratio (1:50) at 37 °C for 16 hours. After that, DTT was added to final concentration 5 mM and then incubated at 50 °C for 30 min, then IAA was added with final concentration 15 mM followed by incubation at room temperature in total darkness for 30 min. The alkylation reaction was terminated by 30 mM cysteine at room temperature for another 30 min. Again Trypsin was added with ratio of trypsin to protein at 1:100 for digestion at 37 °C for 4 hours to ensure the complete digestion. The sample was then fractionated into fractions by high pH reverse-phase HPLC by using Agilent 300Extend C18 column (5 μm particles, 4.6 mm ID, 250 mm length). In brief, peptides obtained were separated into 80 fractions with a gradient of 2% to 60% acetonitrile in 10 mM ammonium bicarbonate (pH 10) for over 80 min first, then they were combined into 18 fractions and dried.

### Affinity Enrichment of Lysine Acetylated and Succinylated Peptides

Tryptic peptides dissolved in NETN buffer were incubated with pre-washed antibody beads (PTM Biolabs) (4 times with NETN buffer and 2 times with ddH_2_O) at 4 °C overnight with gentle shaking. And then, the bound peptides were eluted from the beads with 0.1% TFA, and the eluted fractions were combined and vacuum-dried. The peptides obtained were cleaned with C18 ZipTips (Millipore, MA, USA) according to the manufacturer’s instructions, followed by LC-MS/MS analysis.

### LC-MS/MS Analysis

Three parallel analyses for each fraction were performed. Peptides were dissolved in 0.1% FA (Sigma Fluka, MO, USA), directly loaded onto a reversed-phase pre-column (Acclaim PepMap 100, Thermo, MA, USA). Peptides were separated by using a reversed-phase analytical column (Acclaim PepMap RSLC). The gradient was comprised of a span starting from 6% to 23% solvent B (0.1% FA in 98% ACN) for 24 min, 23% to 35% for 8 min, 80% in 4 min, and at last holding at 80% for another 4 min. All of the above procedures were at a constant flow rate of 280 nL/min on EASY-nLC 1000 UPLC system, and the resulting peptides were analyzed by Q Exactive^TM^ Plus hybrid quadrupole-Orbitrap mass spectrometer (Thermo Fisher, MA, USA).

The peptides were subjected to NSI source, followed by tandem mass spectrometry (MS/MS) in Q Exactive^TM^ Plus (Thermo, MA, USA) coupled online to the UPLC. We detected the intact peptides in the Orbitrap at a resolution of 70,000. Peptides were selected using NCE setting as 30; ion fragments were detected at a resolution of 17,500. A data-dependent procedure that alternated between one MS scan followed by 20 MS/MS scans was applied for the top 20 precursor ions above a threshold ion count of 1.5 E4 in the MS survey scan with 30.0 s dynamic exclusion. The electrospray voltage applied was 2.0 kV. Automatic gain control (AGC) was used to prevent overfilling of the ion trap; 5 E4 ions were accumulated for generation of MS/MS spectra. For MS scans, the m/z scan range was 350 to 1800.

### Data processing

The data obtained was processed using MaxQuant with integrated Andromeda search engine (v.1.4.1.2). Tandem mass spectra were searched against SwissProt_human database concatenated with reverse decoy database. Mass error was set to 10 ppm for precursor ions and 0.02 Da for fragment ions. Carbamidomethylation on Cys was specified as fixed modification and oxidation on Met, acetylation or succinylation on Lys and protein N-terminal were specified as variable modifications. Minimum peptide length was set at 7. False discovery rate (FDR) thresholds for protein, peptides and modification sites were specified at 1%. All the other parameters in MaxQuant were the default values. The Kac and Ksu site localization probabilities were set as >0.75.

### Bioinformatics Methods

Gene Ontology (GO) annotation proteome was derived from the UniProt-GOA database (http://www.ebi.ac.uk/GOA/). For proteins not annotated by UniProt-GOA database (http://www.ebi.ac.uk/GOA/), the InterProScan soft was used to annotated protein’s GO functional based on protein sequence alignment method. Kyoto Encyclopedia of Genes and Genomes (KEGG) database (http://www.genome.jp/kegg/) was used to annotate protein pathway. Online service tools KAAS was used to annotate protein’s KEGG description. KEGG mapper was used to map the annotation results. Functional Annotation Tool of DAVID Bioinformatics Resources 6.7 to identify enriched GO, KEGG pathway and protein domain against the background of Homo sapiens. A two-tailed Fisher’s exact test was employed to test the enrichment of the protein-containing IPI entries against all IPI proteins. Correction for multiple hypothesis testing was carried out using standard false discovery rate control methods. The GO with a corrected *p*-value < 0.05 is considered significant. The protein complex database CORUM was used for protein complex analysis. Corrected *p*-value < 0.05 was considered significant when performing the bioinformatics analysis.

## Additional Information

**How to cite this article**: Zhu, D. *et al*. Crosstalk among proteome, acetylome and succinylome in colon cancer HCT116 cell treated with sodium dichloroacetate. *Sci. Rep*. **6**, 37478; doi: 10.1038/srep37478 (2016).

**Publisher's note:** Springer Nature remains neutral with regard to jurisdictional claims in published maps and institutional affiliations.

## Supplementary Material

Supplementary Information

Supplementary Table S1

Supplementary Table S2

Supplementary Table S2

Supplementary Table S4

Supplementary Table S5

Supplementary Table S6
