# Supplementary material for: Crosstalk among proteome, acetylome and succinylome in colon cancer HCT116 cell treated with sodium dichloroacetate
Source: Sci Rep. 2016 Nov 22;6:37478. doi: 10.1038/srep37478 (PMC5118697; doi:10.1038/srep37478)
Supplement: Supplementary Information [file srep37478-s1.pdf]

Crosstalk among proteome, acetylome and succinylome in colon cancer HCT116 cell line treated with sodium dichloroacetate

Danxi Zhu[1#], Lidan Hou[1#], Bin Hu[2], Hang Zhao[1], Jie Sun[3], Jianhua Wang[4*], Xiangjun Meng[1*]

[1] Department of Gastroenterology, Shanghai Ninth People's Hospital, Shanghai Jiao Tong University School of Medicine, Shanghai 200011, P. R. China.

[2] Department of Gastroenterology, Shanghai General Hospital, Shanghai, 200060, P. R. China.

[3] Department of Gastroenterology, Suzhou Science & Technology Town Hospital, Suzhou, 215000, P. R. China.

[4] Fudan University Shanghai Cancer Center, Fudan University, Cancer institute

Correspondence and requests for materials should be addressed to D.X.Z (zhudx91@126.com, Ph: +86 15000029712) or X.J.M (xiangjunmeng@aliyun.com, Ph: +86 13816170604) or J.H.W (jianhuaw2007@qq.com, Ph: +86 13482192536)

Figure Legends

Figure-S1: Clustering analysis of the quantified proteome based on the functional enrichment. (A) molecular function, (B) cellular compartment, (C) biological process, (D) KEGG pathway, and (E)protein domain.

Figure-S2: (A) Acetylation motifs and conservation of acetylation sites. (B) Succinylation motifs and conservation of succinylation sites. The center K refers to the modified lysine. The enrichment of amino acids in positions around the modified lysine was done by Motif-X software. (C) Heat map of the amino acids upstream and downstream of the acetylated sites shows the relative abundance of the different types of amino acids flanking the modified lysine. (D) Heat map of the amino acids upstream and downstream of the succinylated sites shows the relative abundance of the different types of amino acids flanking the modified lysine.

Figure-S3: (A) crosstalk between global proteome and acetylome. (B) crosstalk between global proteome and succinylome.

Figure-S4: Protein-protein interaction network of (A) acetylome and (B) succinylome.

Figure-S1

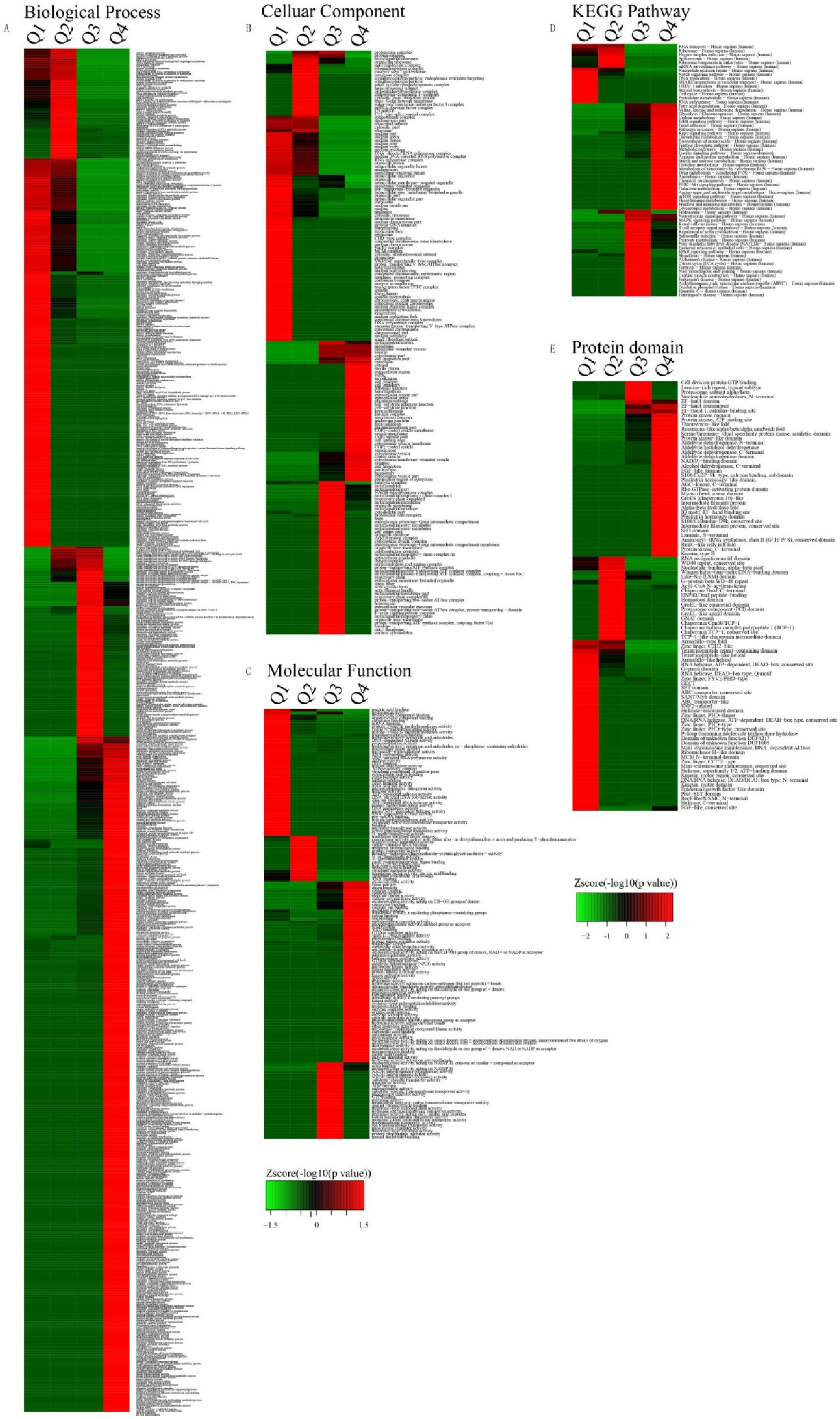
A
Biological Process
Q1 Q2 Q3 Q4
B
Cellular Component
Q1 Q2 Q3 Q4
C
Molecular Function
Q1 Q2 Q3 Q4
Zscore(-log10(p value))
-1.5 0 1.5
D
KEGG Pathway
Q1 Q2 Q3 Q4
E
Protein domain
Q1 Q2 Q3 Q4
Zscore(-log10(p value))
-2 -1 0 1 2

Figure-S2

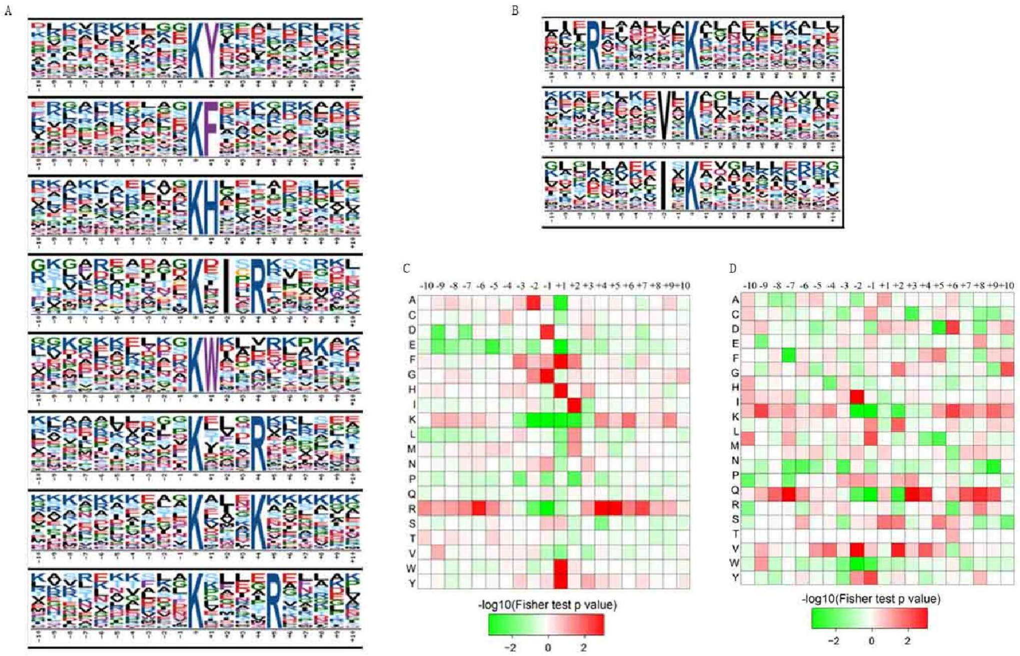

Figure-S3

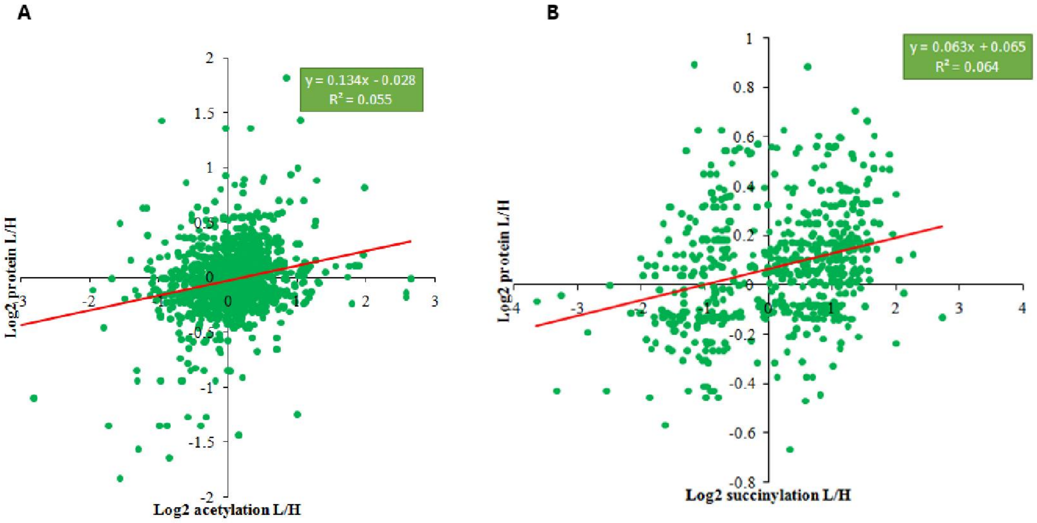

Figure-S4

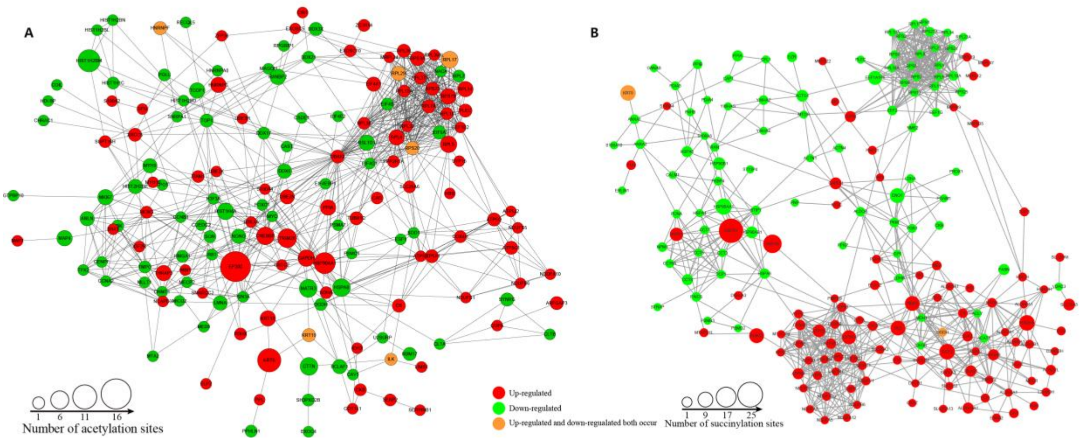
A
B
1 6 11 16
Number of acetylation sites
Up-regulated
Down-regulated
Up-regulated and down-regulated both occur
1 9 17 25
Number of succinylation sites
